# Significance of low ferritin without anaemia in screen‐detected, adult coeliac disease patients

**DOI:** 10.1111/joim.13548

**Published:** 2022-08-04

**Authors:** Marleena Repo, Kalle Kurppa, Heini Huhtala, Liisa Luostarinen, Katri Kaukinen, Laura Kivelä

**Affiliations:** ^1^ Tampere Centre for Child, Adolescent and Maternal Health Research Faculty of Medicine and Health Technology Tampere University Tampere Finland; ^2^ Celiac Disease Research Center Faculty of Medicine and Health Technology Tampere University Tampere Finland; ^3^ Department of Pediatrics Central Finland Central Hospital Jyväskylä Finland; ^4^ The University Consortium of Seinäjoki and Seinäjoki Central Hospital Seinäjoki Finland; ^5^ Faculty of Social Sciences Tampere University Tampere Finland; ^6^ Department of Neurology Päijät‐Häme Central Hospital Lahti Finland; ^7^ Department of Internal Medicine Tampere University Hospital Tampere Finland; ^8^ Children's Hospital and Pediatric Research Center University of Helsinki and Helsinki University Hospital Helsinki Finland

**Keywords:** coeliac disease, ferritin, follow‐up, symptoms, quality of life

## Abstract

**Background:**

Low ferritin without anaemia has been linked to adverse health effects.

**Objectives:**

To investigate the prevalence and clinical significance of low ferritin in screen‐detected coeliac disease.

**Methods:**

Seventy‐six screen‐detected coeliac disease patients were enrolled in the prospective collection of comprehensive clinical, laboratory and histological data at diagnosis and after 1–2 years on a gluten‐free diet (GFD). All variables were compared between patients with different ferritin levels.

**Results:**

At coeliac disease diagnosis, six patients had anaemia. Of the 70 nonanaemic patients, ferritin levels were <15 μg/L in 21%, 15–29 μg/L in 19%, 30–99 μg/L in 36% and ≥100 μg/L in 24%. Those with lower ferritin were more often females, had lower body mass index, haemoglobin and villous height–crypt depth ratio and also had higher intra‐epithelial lymphocyte CD3+ levels in duodenal biopsies. The groups did not differ in neurological or gastrointestinal symptoms, health‐related quality of life, bone mineral density, liver values, vitamin, albumin or coeliac autoantibody levels or the prevalence of comorbidities. Median ferritin levels increased from 41.5 μg/L to 86.0 μg/L on GFD (*p* < 0.001). Ferritin remained <30 μg/L in 21% of patients but was not associated with dietary compliance, nor was any correlation between changes in ferritin and quality of life, gastrointestinal symptoms, autoantibody levels or degree of histological damage detected.

**Conclusion:**

Decreased ferritin is a frequent finding in screen‐detected coeliac disease and may not be fully restored on a GFD. However, low ferritin levels are not associated with more severe symptoms or poorer quality of life.

## Graphical abstract

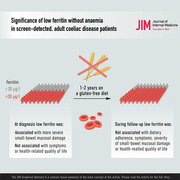

## Introduction

Reduced iron stores without anaemia—characterised by low ferritin and normal haemoglobin levels—are known to be common in growing children, menstruating and pregnant women and regular blood donors [1, 2, 3]. However, the exact definition of low ferritin and its clinical significance without anaemia has been under intense debate [4, 5, 6, 7]. Recently, it has been suggested that this entity may be associated with unspecific symptoms such as fatigue and poorer quality of life [8, 9, 10, 11, 12]. However, many of these studies have been conducted in selected—by definition symptomatic—patient groups, for example, in those with restless leg syndrome, chronic heart failure, fibromyalgia syndrome or hypothyroidism [8, 9, 10, 11], and population‐based studies are rare [12, 13]. More data about the significance of ferritin levels in other chronic conditions and in apparently asymptomatic individuals are needed.

Coeliac disease is a chronic, immune‐mediated condition where dietary gluten induces small‐bowel mucosal damage and systemic consequences in genetically susceptible individuals [14]. Patients may suffer from gastrointestinal symptoms but also from extraintestinal manifestations, including iron deficiency and anaemia [15, 16]. Some patients are asymptomatic and present only with laboratory abnormalities. Anaemia in coeliac disease seems to be associated with more severe clinical features compared to nonanaemic patients or those presenting with diarrhoea [17, 18, 19, 20, 21, 22], but the prevalence and significance of iron deficiency without anaemia have rarely been studied [23, 24]. Usually, initiation of a strict gluten‐free diet (GFD) results in improved haemoglobin levels parallel with alleviation of small‐bowel mucosal damage [17, 22], but the response of ferritin levels to the dietary treatment, particularly in patients with initially normal haemoglobin, is less clear.

In this prospective study, we aimed to evaluate the prevalence of low ferritin without anaemia defined by different thresholds in adult screen‐detected coeliac disease patients. Additionally, we studied possible associations between ferritin levels and comprehensive clinical, histopathological and laboratory characteristics as well as the changes in ferritin levels on a GFD.

## Material and methods

### Patients and study design

The study was conducted in Tampere University, Tampere University Hospital and Päijät‐Häme Central Hospital. Inclusion criteria for all study patients were age above 18 years, recently diagnosed coeliac disease (positive coeliac autoantibodies and histologically verified diagnosis, defined as Marsh III degree small‐bowel mucosal damage) and availability of serum ferritin level at diagnosis. Exclusion criteria was initiation of a GFD before the measurement of the coeliac autoantibodies and other laboratory results.

The patients were diagnosed with coeliac disease in the population or at‐risk group screening. In the ‘Päijät‐Häme group’, a randomly selected cohort of 4272 elderly adults representing the Finnish population above 50 years of age was invited to coeliac disease screening, and 2815 (66%) consented to participate [25]. Of these, 37 had positive tissue transglutaminase antibodies (TGA) and fulfilled the study criteria. In the ‘Tampere group’, coeliac disease patients were recruited from patient support groups and by newspaper advertisements, and were asked to invite their relatives to participate in the study. In total, 3031 risk‐group members of all ages participated in the screening [26]. Of these, 39 were endomysium antibody (EmA) positive and fulfilled the above‐mentioned and additional—no ongoing/planned pregnancy, obvious clinical symptoms, severe concurrent illness or immunosuppressive medication—study criteria. All recently diagnosed patients were evaluated at coeliac disease diagnosis and followed up for 1–2 years on a GFD.

Of the 76 patients included in the study, six (8%) had anaemia at coeliac disease diagnosis and were excluded from further analyses. The remaining 70 nonanaemic patients comprised the final study cohort.

### Ethical considerations

The study protocol was approved by the regional Ethics Committees of Päijät‐Häme Central Hospital and Tampere University Hospital. Patients were informed about the screening protocol, coeliac disease diagnosis and its treatment before screening, and written informed consent was obtained from all participants. The study was conducted according to the Helsinki Declaration [27].

### Data collection

Data were collected prospectively at the time of coeliac disease diagnosis and after 1–2 years on a GFD, including demographic and clinical characteristics, general health, quality of life, bone health and body composition, severity of small‐intestinal mucosal damage, coeliac antibody levels, blood haemoglobin, serum ferritin and other laboratory values. Self‐reported adherence to a GFD was evaluated on a follow‐up visit.

The presence of any symptoms, chronic comorbidities, history of fractures and family history of coeliac disease was assessed at coeliac disease diagnosis through interviews conducted by the study physicians/nurses. In addition, the presence of neurological symptoms, including numbness, headache, migraine, unspecific pain/stinging and ataxia was evaluated in the ‘Päijät‐Häme group’.

All patients completed validated questionnaires for gastrointestinal symptoms and health‐related quality of life. The Gastrointestinal Symptom Rating Scale (GSRS) is a 15‐item questionnaire where the severity of diarrhoea, indigestion, constipation, abdominal pain and gastroesophageal reflux symptoms are estimated on a Likert scale from one (no symptoms) to seven (very severe symptoms) [28]. The total score is calculated as a mean of all 15 items and values for subcategories by means of related items. The Psychological General Well Being (PGWB) questionnaire consists of 22 questions, including both negative and positive affective states divided into anxiety, depressed mood, positive well‐being, self‐control, general health and vitality [29]. Every question is scored from one to six, with higher scores denoting better quality of life. The total score is calculated as the sum of all scores, and subscores as the sums of the scores of 2–4 related questions.

Body mass index (BMI) was computed as weight/height^2^ (kg/m^2^) for all patients. Bone mineral density (BMD) was measured by dual‐energy X‐ray absorptiometry in the lumbar spine and right femoral neck (in the ‘Päijät‐Häme group’ by GE Medical Systems, LUNAR, UK, and in the ‘Tampere group’ by Lunar Prodigy Advance, GE Healthcare, Waukesha, WI, USA). BMD values were expressed as T‐scores, which compare bone density to that in healthy 30‐year‐olds of the same sex and as age‐ and sex‐matched Z‐scores. Body fat percentage was measured in the ‘Tampere group’ patients by dual‐energy X‐ray absorptiometry (Lunar Prodigy Advance, GE Healthcare, Waukesha, WI, USA).

An upper gastrointestinal endoscopy with 3–6 duodenal biopsy specimens was performed at coeliac disease diagnosis and at a follow‐up visit after 1–2 years on a GFD. Villous height–crypt depth ratio (VH:CrD) was measured using paraffin‐embedded, haematoxylin–eosin stained and correctly oriented biopsy specimens. Lower VH:CrD indicates more severe villous atrophy [30]. In addition, total intra‐epithelial lymphocyte (IEL) densities and their changes during follow‐up were counted from the biopsies, and more specific CD3+ IEL densities (reference value [Rf] <37 cells/mm) were evaluated at diagnosis [31].

After study enrolment, both TGA and EmA were evaluated in all patients at coeliac disease diagnosis and during follow‐up. TGA were analysed by enzyme‐linked immunosorbent assay (Celikey; Phadia, Freiburg, Germany) according to the manufacturer's instructions. Values ≥5.0 kU/L were considered elevated. EmA were analysed by an indirect immunofluorescence method using human umbilical cord as a substrate. A dilution greater than or equal to 1:5 was considered positive and further diluted up to 1:4000 or negative [32].

Other laboratory values were measured using standard laboratory methods. Serum ferritin, blood haemoglobin (Rf, men 134–167 g/L; women 117–155 g/L), serum vitamin B12 (Rf 150–740 pmol/L), red blood cell folate (Rf 200–700 nmol/L) and serum ionized calcium (Rf 1.20–1.35 mmol/L) levels were analysed in all patients. Additionally, plasma albumin (Rf 36–48 g/L) and plasma parathormone (Rf 1.6–6.9 pmol/L) levels were measured in the ‘Tampere group’, and plasma phosphate (Rf men 0.71–1.23 mmol/L; women 0.76–1.41 mmol/L) and serum vitamin D25 (Rf 50–75 nmol/L) levels in the ‘Päijät‐Häme group’ [33].

Study patients were divided into four groups according to their serum ferritin levels at coeliac disease diagnosis: <15 μg/L, 15–29 μg/L, 30–99 μg/L or ≥100 μg/L. These cut‐offs for the study groups were selected because they are the most commonly used definitions for iron deficiency based on serum ferritin levels [6]. A ferritin level ≥100 μg/L was taken as a treatment target, as has frequently been recommended [6, 34].

To ensure a strict GFD, patients received counselling from a dietitian at coeliac disease diagnosis and after 1 year on the diet. Adherence to the diet was defined as strict (no lapses) or nonadherent according to patients’ reports.

### Statistics

Study variables were described as numbers and percentage distributions or as medians and quartiles, as most quantitative variables were found to be skewed by the Shapiro–Wilk method. Statistical analyses were conducted using χ^2^ or Fisher's exact test for categorical variables, and with the Mann–Whitney U, Kruskal–Wallis or Wilcoxon test for continuous variables. Correlations between continuous variables were calculated using Spearman's rank correlation. Significant differences between the four ferritin groups were adjusted for age and sex with linear regression. A *p*‐value <0.05 was considered statistically significant. All statistical analyses were performed using IBM SPSS Statistics version 26 (IBM Corp, Armonk, NY, USA).

## Results

The median ferritin levels at diagnosis were 41.5 (interquartile range [IQR] 18.5–99.0) μg/L in all nonanaemic patients, 31.0 (IQR 10.5–69.5) μg/L in the ‘Päijät‐Häme group’ and 73.0 (IQR 19.5–110.0) μg/L in the ‘Tampere group’. Of the 70 nonanaemic coeliac disease patients, 21% had ferritin <15 μg/L, 19% between 15 and 29 μg/L, 36% between 30 and 99 μg/L and 24% ≥100 μg/L at diagnosis. Low ferritin levels were associated with female sex and lower BMI (Table 1). Difference in BMI remained significant after adjusting for age and sex. The four groups did not differ significantly in age at diagnosis, family history of coeliac disease, comorbidities, prevalence of fractures, body composition or BMD (Table 1).

**Table 1:** Demographic characteristics, comorbidities, body composition and bone health in 70 screen‐detected and nonanaemic patients with different ferritin levels at the time of coeliac disease diagnosis

	Ferritin at coeliac disease diagnosis				
	<15 μg/L, *n* = 15	15–29 μg/L, *n* = 13	30–99 μg/L, *n* = 25	≥100 μg/L, *n* = 17	
	%	%	%	%	*P*‐value
Women	80	46	56	12	**0.001**
Family history of coeliac disease	53	75	67	82	0.345
Chronic comorbidity^a^	77	69	60	35	0.101
Unspecified fractures	20	46	23	42	0.339

	Median (IQR)	Median (IQR)	Median (IQR)	Median (IQR)	
Age at screening, years	56 (42–67)	58 (55–67)	54 (34–58)	46 (42–55)	0.086
Body mass index, kg/m^2^	25 (23–28)	24 (22–28)	25 (24–30)	29 (26–30)	**0.011** ^e^
Body fat percentage^b^	33 (26–38)	33 (26–41)	30 (22–38)	31 (28–35)	0.864
T‐score L2‐L4^c^	−0.3 (−1.8 to 1.4)	−0.8 (−2.0 to 0.5)	−0.6 (−1.4 to 1.0)	−0.1 (−1.5 to 0.8)	0.889
Z‐score L2‐L4^d^	0.6 (−0.5 to 1.6)	0.7 (−1.3 to 1.5)	−0.1 (−0.7 to 1.7)	−0.2 (−1.9 to 1.3)	0.489
T‐score F neck^c^	−0.4 (−2.2 to 0.0)	−1.0 (−2.2 to −0.1)	−0.5 (−1.4 to 0.2)	−0.6 (−1.7 to −0.1)	0.482
Z‐score F neck^d^	0.0 (−0.6 to 1.0)	0.1 (−0.8 to 0.7)	0.0 (−0.8 to 0.5)	−0.4 (−0.9 to 0.3)	0.542

*Note*: Values in boldface denote statistically significant difference.

Abbreviation: IQR, interquartile range.

^a^For example, hypothyroidism, hyperlipidaemia, hypertension, psoriasis, coronary artery disease, neurological disease, sarcoidosis, endometriosis and type 1 or 2 diabetes. Allergies, lactose intolerance, atopic dermatitis and unspecified musculoskeletal symptoms were excluded. Data were available for >90% of patients, except on body fat percentage data were available in 51% of the patients.

^b^Data were available in 51% of the patients.

^c^Comparison of bone density to healthy 30‐year‐olds of the same sex.

^d^Comparison of bone density to an average person of the same age and sex.

^e^Remained significant after adjusting for age and sex.

Patients with ferritin <15 μg/L and 30–99 μg/L at diagnosis reported lower vitality than those with ferritin 15–29 μg/L and ≥100 μg/L, but the difference was not significant after adjusting for age and sex (Table 2). Other aspects and overall health‐related quality of life measured with PGWB were comparable between the groups. Prevalence of symptoms in general, specific neurological symptoms (Fig. S1) or severity of the gastrointestinal symptoms measured with GSRS (Table 2) did not differ between the study groups.

**Table 2:** Health‐related quality of life and gastrointestinal symptoms in 70 screen‐detected and nonanaemic patients with different ferritin levels at the time of coeliac disease diagnosis

	Ferritin at coeliac disease diagnosis				
	<15 μg/L, *n* = 15	15–29 μg/L, *n* = 13	30–99 μg/L, *n* = 25	≥100 μg/L, *n* = 17	
	Median (IQR)	Median (IQR)	Median (IQR)	Median (IQR)	*P*‐value
**PGWB** ^a^					
Total	104 (96–112)	118 (107–123)	104 (89–115)	110 (97–119)	0.077
Vitality	18 (16–20)	21 (19–22)	17 (14–21)	20 (17–22)	0.028^c^
Positive well‐being	17 (16–20)	20 (17–21)	17 (14–19)	17 (17–21)	0.105
Anxiety	24 (20–26)	27 (25–27)	25 (21–26)	25 (22–28)	0.178
Depressive mood	18 (15–18)	18 (17–18)	18 (15–18)	18 (16–18)	0.762
General health	14 (13–15)	16 (14–17)	13 (10–17)	14 (13–16)	0.182
Self control	16 (14–17)	17 (16–17)	16 (14–17)	17 (16–18)	0.223
**GSRS** ^b^					
Total	1.9 (1.3–2.5)	1.7 (1.2–2.2)	1.8 (1.4–2.6)	1.7 (1.2–2.2)	0.633
Constipation	1.3 (1.0–3.3)	1.3 (1.0–2.3)	1.7 (1.0–2.0)	1.0 (1.0–1.8)	0.623
Diarrhoea	1.3 (1.0–1.7)	1.2 (1.0–1.7)	1.7 (1.0–2.8)	1.3 (1.0–2.3)	0.495
Indigestion	2.5 (1.0–3.3)	2.3 (1.6–3.2)	2.5 (1.5–3.7)	2.5 (1.5–2.9)	0.927
Pain	1.7 (1.7–2.0)	1.5 (1.0–2.3)	1.7 (1.3–2.6)	1.7 (1.2–2.0)	0.813
Reflux	1.0 (1.0–2.0)	1.0 (1.0–2.5)	1.0 (1.0–1.5)	1.0 (1.0–1.5)	0.990

*Note*: Data were available on ≥90% of patients.

Abbreviations: GSRS, Gastrointestinal Symptom Rating Scale; IQR, interquartile range; PGWB, Psychological General Well Being questionnaire.

^a^Higher scores denote better health‐related quality of life.

^b^Higher scores denote more severe symptoms.

^c^Not significant after adjusting for age and sex.

**Fig. 1:**
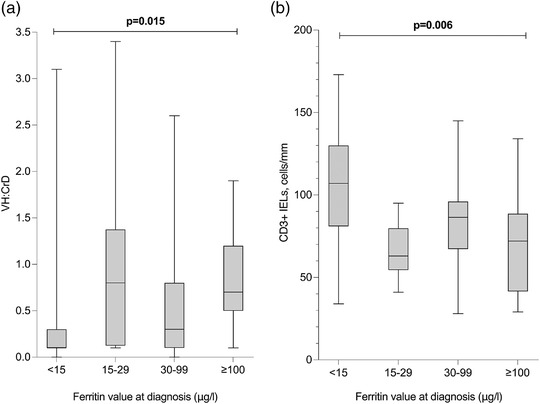
(a) Small‐bowel mucosal villous height–crypt depth ratio (VH:CrD) and (b) density of CD3+ intra‐epithelial lymphocytes (IELs) in 70 screen‐detected and nonanaemic patients with different ferritin levels at the time of coeliac disease diagnosis. The values are presented as medians with quartiles (boxes) and range (whiskers). Data were available on 97% of patients.

Patients with ferritin <15 μg/L at diagnosis had lower VH:CrD and higher density of CD3+ IELs than those with higher ferritin levels, both in crude analysis and after adjusting for age and sex (Fig. 1). Ferritin <15 μg/L was also associated with lower haemoglobin, which remained significant after adjustments, whereas albumin levels were lower in crude but not in adjusted analyses (Table 3). Other laboratory results including coeliac antibody levels were comparable between the groups (Table 3).

**Table 3:** Coeliac antibody levels, iron metabolism and other laboratory values in 70 screen‐detected and nonanaemic patients with different ferritin levels at the time of coeliac disease diagnosis

	Ferritin at coeliac disease diagnosis				
	<15 μg/L, *n* = 15	15–29 μg/L, *n* = 13	30–99 μg/L, *n* = 25	≥100 μg/L, *n* = 17	
	Median (IQR)	Median (IQR)	Median (IQR)	Median (IQR)	*P*‐value
**Coeliac antibody levels**					
Serum EmA, titre	1:100 (1:5–1:500)	1:200 (1:100–1:1000)	1:200 (1:5–1:500)	1:100 (1:5–1:200)	0.402
Serum TGA, kU/I	33 (8–93)	30 (13–64)	21 (6–57)	11 (6–25)	0.213
**Iron metabolism**					
Blood haemoglobin, g/L	134 (120–138)	141 (137–146)	146 (137–153)	150 (147–162)	**<0.001** ^d^
Serum B12‐vitamin, pmol/L	271 (188–368)	301 (232–389)	328 (230–427)	318 (252–433)	0.405
RBC folate, nmol/L	280 (154–473)	381 (255–642)	386 (270–592)	354 (291–540)	0.337
**Other**					
Plasma albumin, g/L^a^	38 (35–40)	39 (37–42)	43 (41–44)	41 (38–42)	0.008^e^
Serum Ca‐ion, mmol/L	1.23 (1.22–1.25)	1.25 (1.23–1.28)	1.26 (1.24–1.29)	1.26 (1.23–1.29)	0.080
Plasma phosphate, mmol/L^b^	0.92 (0.79–1.05)	0.97 (0.74–1.10)	0.98 (0.88–1.11)	0.89 (0.82–0.96)	0.562
Plasma PTH, pmol/L^a^	6.4 (3.5–7.9)	5.4 (3.2–7.7)	4.4 (3.1–5.8)	4.4 (4.1–6.3)	0.465
Serum vitamin D25, nmol/L^c^	45 (33–60)	47 (35–76)	39 (37–52)	42 (41–55)	0.725

*Note*: Data were available on >90% of patients, except where noted.

Values in boldface denote statistically significant difference.

Abbreviations: Ca‐ion, ionized calcium; EmA, endomysium antibodies; IQR, interquartile range; PTH, parathormone; RBC, red blood cell; TGA, tissue transglutaminase antibodies.

^a^Data were available on 53% of patients.

^b^Data were not available on 47% of patients.

^c^Data were not available on 44% of patients.

^d^Difference remained significant after adjusting for age and sex.

^e^Difference was not significant after adjusting for age and sex.

**Fig. 2:**
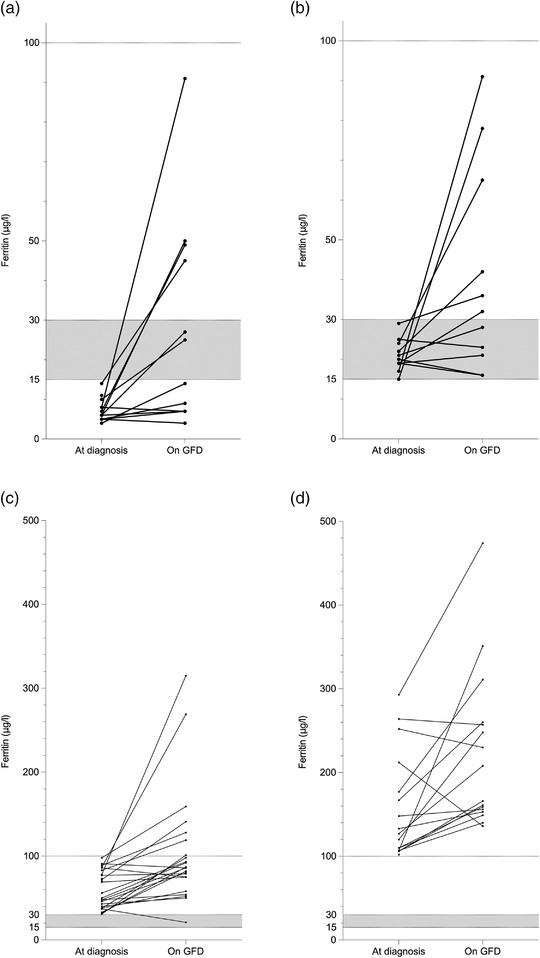
Changes in ferritin levels after 1–2 years on a gluten‐free diet (GFD) in patients with initial ferritin levels (a) <15 μg/L, (b) 15–29 μg/L, (c) 30–99 μg/L and (d) >100 μg/L at the time of coeliac disease diagnosis. Ferritin cut‐offs <15 μg/L, <30 μg/L and <100 μg/L are the most used definitions for iron deficiency based on serum ferritin levels, and ferritin ≥100 μg/L a treatment target. Data were available on 89% of patients. Note the different scale on the y‐axis in a–b versus c–d.

Follow‐up data on ferritin values after 1–2 years on a GFD were available for 87% of the patients. The median levels increased significantly from baseline (41.5 [IQR 18.5–99.0] μg/L vs. 86.0 [IQR 39.0–154.5] μg/L, *p* < 0.001). At the patient level, ferritin increased in 64%, remained within 10 μg/L of the original value in 20% and decreased in 16% (Fig. 2). Ferritin ≥100 μg/L was not reached by any of those with ferritin <30 μg/L at diagnosis, and the level remained <30 μg/L in 21% and <15 μg/L in 8% of the patients (Fig. 2). In those with ferritin <30 μg/L at diagnosis, the change in ferritin levels on a GFD did not correlate with the change in VH:CrD, the density of total IELs or in PGWB or GSRS scores (data not shown).

In a separate analysis, patients with follow‐up ferritin <30 μg/L were more often women (77% vs. 40%, *p* = 0.017) than those with levels ≥30 μg/L, whereas there was no difference in the median age (48 vs. 55 years, *p* = 0.645), dietary adherence (strict diet 69% vs. 77%, *p* = 0.718), presence of chronic comorbidities (46% vs. 60%, *p* = 0.387) or persistent symptoms (0% vs. 9%, *p* = 0.564), VH:CrD, density of total IELs or coeliac antibody levels (median 1 vs. 0 kU/L, *p* = 0.575; EmA 1:<5 vs. 1:<5 titre, *p* = 0.885) or in PGWB or GSRS scores, body composition or BMD (data not shown).

## Discussion

We found ferritin <15 μg/L to be present in 21% and <30 μg/L in 40% of the screen‐detected and nonanaemic coeliac disease patients. Low ferritin values were more common in females and in those with lower, although normal, haemoglobin levels. To the best of our knowledge, the prevalence of low ferritin without anaemia in coeliac disease has not been previously reported. In studies including clinically found anaemic patients, the figures at diagnosis have varied between 14% and 82% [20, 35, 36, 37, 38, 39]. The difference between the studies may be due to varying definitions of low ferritin (12–30 μg/L) and by differences in study population characteristics, such as sex distribution, haemoglobin levels and severity of the disease. In general, ferritin levels are lower in females than in males [40] and reflect the body iron stores [4]. Furthermore, in countries such as India [20], iron deficiency is a common finding in the general population, in contrast to Western countries [41]. Clinically detected patients have sought medical help for their symptoms, resulting in the diagnosis of coeliac disease, in contrast to screen‐detected patients, who might have been found even before the development of symptoms, malabsorption and nutritional deficiencies. This, together with diagnosed anaemia in some of the clinically found patients, likely explains the more common iron deficiency in the studies focusing on this patient group. However, the present results suggest that low ferritin values are also common in otherwise asymptomatic patients.

Besides female sex, lower ferritin levels were associated with lower BMI and with more advanced histological damage and inflammation measured by VH:CrD and density of CD3+ IELs. However, ferritin was not associated with serology. A positive correlation between ferritin and BMI has previously been reported in young males and male blood donors [42, 43], but not in coeliac disease [38]. Studies focusing on anaemic coeliac disease patients have also reported low ferritin to be associated with more severe mucosal damage [17, 18, 19, 20, 21, 22, 23], but—in contrast to our results—also with higher levels of serum autoantibodies [17, 18, 19, 22]. These findings suggest an association between the severity of duodenal damage and iron absorption, but a more complex link between antibody production and iron homeostasis.

Low ferritin levels were not associated with the presence or severity of symptoms or health‐related quality of life at diagnosis. Furthermore, the change in ferritin levels or follow‐up values were not associated with alleviation of histological damage, autoantibody levels, symptom persistence or quality of life. The data so far on nonanaemic coeliac disease patients with low ferritin are limited, whereas patients with anaemia have reported more severe gastrointestinal and extraintestinal symptoms than those with normal haemoglobin [17, 19, 22]. Additionally, nonanaemic iron deficiency has been associated with neurological and psychological symptoms in menstruating females, male municipal employees and in patients with chronic heart failure, fibromyalgia, hypothyroidism and restless legs syndrome [8, 9, 10, 11, 12]. Regarding change in ferritin level, higher values reportedly have a positive effect on fatigue and neurological symptoms in premenstrual women [44]. It is important to realise that iron deficiency anaemia develops only after the body iron stores are depleted [45]. This may also have occurred in our patients if gluten‐containing diet was continued, and one could speculate whether it would have resulted in the development of symptoms or other problems.

Ferritin levels increased in most patients during 1–2 years on a GFD, although the target level of >100 μg/L was seldom reached. The latter is in line with previous evidence that normalization of haemoglobin and ferritin levels may take more than a year in both screen‐detected and clinically found coeliac disease patients [17, 35, 46], indicating that longer follow‐up may be needed. Other explanations for persistently low ferritin could be poor dietary iron supply, inability to change dietary habits especially in the elderly population or the presence of some co‐existing chronic condition. Of note, in some patients, the initial exceptionally high ferritin value actually decreased during dietary treatment, suggesting that the hyperferritinemia could have been caused by some inflammatory process, as ferritin also acts as an acute‐phase protein [47]. Of note, Harper et al. reported GFD to result in decreased ferritin levels in coeliac disease patients with high baseline levels [48]. The often‐slow improvement in ferritin levels on a GFD supports careful monitoring and consideration of additional iron supplementation.

The main strengths of our study are the prospective study design and availability of comprehensive medical data, as well as the use of validated questionnaires on gastrointestinal symptoms and psychological well‐being. Screen‐detected coeliac disease enabled focusing on possibly iron‐deficient patients who do not yet have anaemia or clinical symptoms and thus fewer confounding factors. Limitations include the rather short follow‐up time and lack of data on nutrition, and the possible presence of co‐existing inflammatory diseases, iron supplementation and medications that may affect iron metabolism. Additionally, measurement of the study parameters was somewhat unsystematic, and dietary adherence was assessed based on self‐reporting instead of validated questionnaires. Also, the use of different screening outcomes (TGA vs. EmA) in the two study cohorts might have affected the detection rate. Selection bias cannot be ruled out, as symptomatic patients with possibly lower ferritin could have been more prone to participate, and we were not able to compare our patients to noncoeliac controls. Finally, the generalizability of our results—for example, in countries where iron deficiency is more common—is limited.

To conclude, low ferritin without anaemia is a common finding in screen‐detected and even asymptomatic coeliac disease, especially in women. It is associated with lower BMI and with more severe duodenal mucosal damage, but not with more severe symptoms or poorer quality of life. In light of these results, it remains debatable whether ferritin levels should be routinely measured in coeliac disease, as they were not associated with the health outcomes. Although the levels improved on a GFD, many patients also presented with low ferritin during 1‐to‐2‐year follow‐up. When iron deficiency is detected, the follow‐up period should be long enough to ascertain the normalization of the values.

## Funding

This study was supported by the Competitive State Research Financing of the Expert Area of Tampere University Hospital, the Päivikki and Sakari Sohlberg Foundation, the Maire Rossi Foundation, the Maud Kuistila Foundation, the Mary and Georg Ehrnrooth Foundation, the Paulo Foundation, the Emil Aaltonen Foundation, the Finnish Coeliac Society, the Sigrid Juselius Foundation, the Foundation for Pediatric Research, the Finnish Cultural Foundation and the Academy of Finland.

## Conflict of interest

Marleena Repo, Laura Kivelä, Katri Kaukinen and Kalle Kurppa have received personal lecture fees from the Finnish Coeliac Society outside the submitted work, and Laura Kivelä, Katri Kaukinen and Kalle Kurppa serve as members of the advisory committee of the Finnish Coeliac Society.

## Author contributions

Marleena Repo: Conceptualization; Formal analysis; Investigation; Methodology; Validation; Visualization; Writing – original draft. Kalle Kurppa: Conceptualization; Data curation; Funding acquisition; Investigation; Methodology; Validation; Visualization; Writing – review and editing. Heini Huhtala: Conceptualization; Formal analysis; Methodology; Visualization; Writing – review and editing. Liisa Luostarinen: Conceptualization; Investigation; Writing – review and editing. Katri Kaukinen: Conceptualization; Data curation; Funding acquisition; Investigation; Methodology; Project administration; Validation; Visualization; Writing – review and editing. Laura Kivelä: Conceptualization; Data curation; Formal analysis; Funding acquisition; Investigation; Methodology; Validation; Visualization; Writing – original draft.

## Supporting information


**Figure S1**: Prevalence of neurological symptoms in 32 screen‐detected and nonanaemic patients with different ferritin levels at the time of coeliac disease diagnosis.
