# Supplementary figures and images for: Significance of low ferritin without anaemia in screen‐detected, adult coeliac disease patients
Source: J Intern Med. 2022 Aug 4;292(6):904–14. doi: 10.1111/joim.13548 (PMC9805163; doi:10.1111/joim.13548)

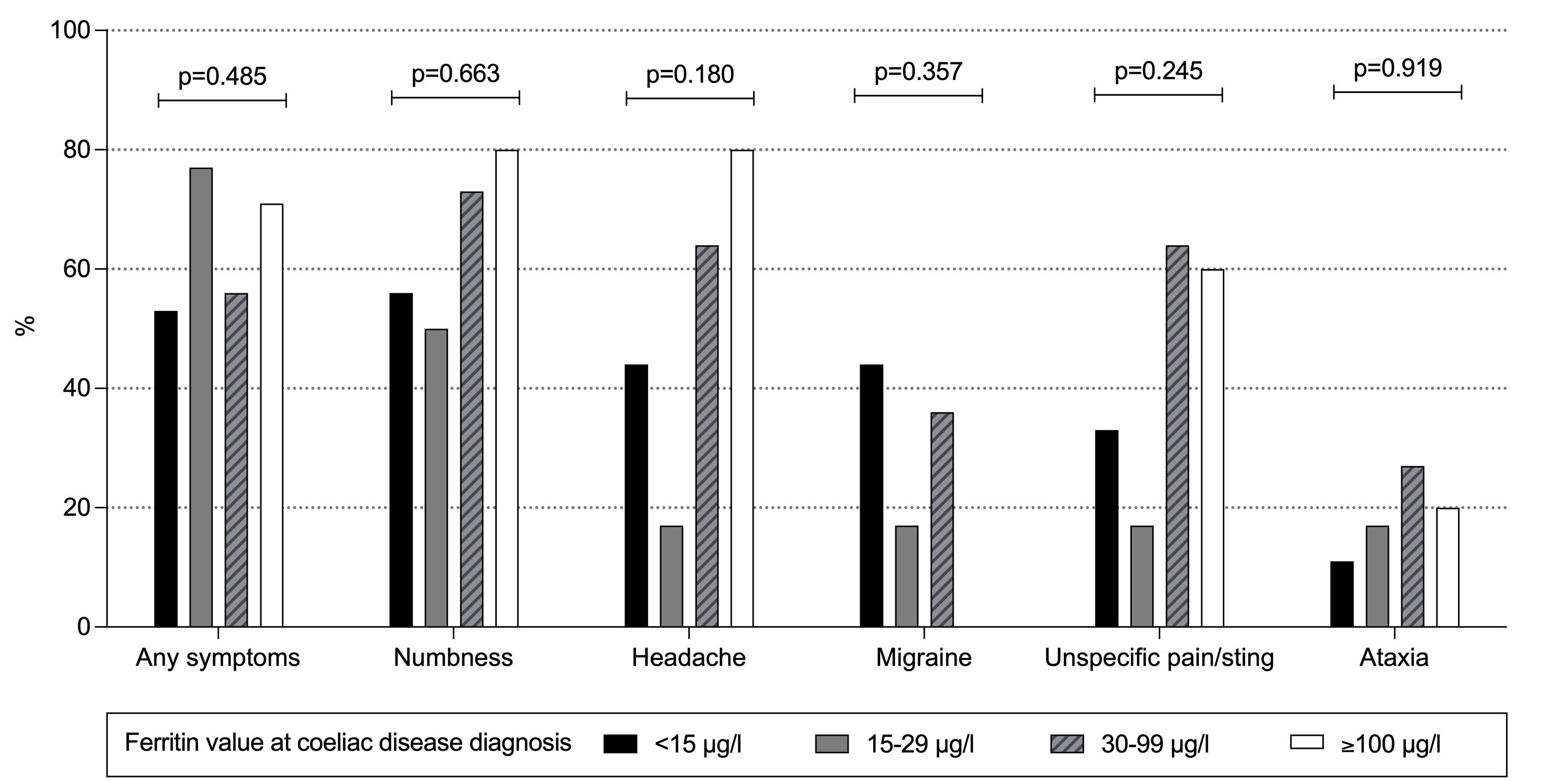

Supplement: Supplementary file 1 — Figure S1: Prevalence of neurological symptoms in 32 screen‐detected and nonanaemic patients with different ferritin levels at the time of coeliac disease diagnosis. [file JOIM-292-904-s001.tiff]
